# Late radiation-induced cardiovascular disease after childhood rhabdomyosarcoma: a case report

**DOI:** 10.1093/ehjcr/ytag488

**Published:** 2026-07-09

**Authors:** Tuan Hoang Nguyen, Takamitsu Nakamura, Akira Sato

**Affiliations:** Department of Cardiovascular Medicine, University of Yamanashi, Faculty of Medicine, 1110 Shimokato, Chuo 409-3898, Japan; Department of Cardiovascular Medicine, University of Yamanashi, Faculty of Medicine, 1110 Shimokato, Chuo 409-3898, Japan; Department of Cardiovascular Medicine, University of Yamanashi, Faculty of Medicine, 1110 Shimokato, Chuo 409-3898, Japan

**Keywords:** Cardiotoxicity, Case report, Cellular senescence, Fibrosis

## Abstract

**Background:**

Radiation-induced cardiovascular disease may appear decades after cancer therapy and can involve multiple vascular, valvular, myocardial, and conduction structures of the heart. Management is particularly challenging when prior irradiation results in a hostile chest environment and impaired wound healing.

**Case summary:**

A 31-year-old woman with a history of childhood right forearm rhabdomyosarcoma treated with resection, chemotherapy, and radiotherapy presented with recurrent syncope. Computed tomography angiography revealed asymmetric growth of the right-sided arteries, leading to a transient ischaemic attack. Further cardiovascular evaluation revealed multiple concomitant cardiovascular disorders, including valvular heart disease, high-grade atrioventricular block, bradycardia, and heart failure. Permanent pacemaker implantation and right-to-left axillary artery bypass prevented further syncopal episodes; however, heart failure continued to worsen. Two years later, she was hospitalized with severe heart failure and dyspnoea, and echocardiography revealed aortomitral continuity calcification and elevated filling pressure. She subsequently underwent the Bentall procedure, tricuspid valve replacement, and coronary artery bypass grafting for advanced coronary stenosis. However, her postoperative course was complicated by pleural effusion and haemoptysis, and she eventually died of multiorgan dysfunction.

**Discussion:**

This case illustrates the late cardiovascular effects of radiation therapy. Radiation-induced endothelial injury, fibrosis, and maladaptive wound-healing responses may contribute to delayed cardiovascular damage and poor postoperative recovery. In such patients, surgical decision-making should be multidisciplinary, and long-term follow-up is essential.

Learning points

- Childhood cancer radiotherapy may lead to senescence of healthy stem cells.
- Further interventions, including surgery, should be approached with caution and guided by multidisciplinary decision-making.
- Screening for complications in organs within the irradiated field deserves careful attention, as late effects may become apparent decades after radiation therapy.

## Introduction

The late effects of radiation therapy on cardiovascular diseases may accumulate and become apparent long after treatment completion. These late effects, namely tissue reactions, are not determined solely by irradiation but may be modified after exposure. The absorbed dose threshold for circulatory disease may be as low as 0.5 Gy.^1^ However, the underlying mechanisms remain unclear, and guidance on diagnosis and management is limited.

## Summary figure

Clinical timeline. † Left internal thoracic artery to the left anterior descending artery and gastroepiploic artery to the posterior descending and posterolateral branches. The timeline figure shows the clinical course, management, and interventions used in this case. Abbreviations: AV block, atrioventricular block; CABG, coronary artery bypass grafting; CVD, cardiovascular disease; CTA, computed tomography angiography; E/e’, ratio of the early diastolic mitral inflow velocity to early diastolic mitral annular tissue velocity; ICU, intensive care unit; LV, left ventricular; RCA, right coronary artery; VAC, vincristine sulphate, actinomycin-D, and cyclophosphamide.

**Figure:**
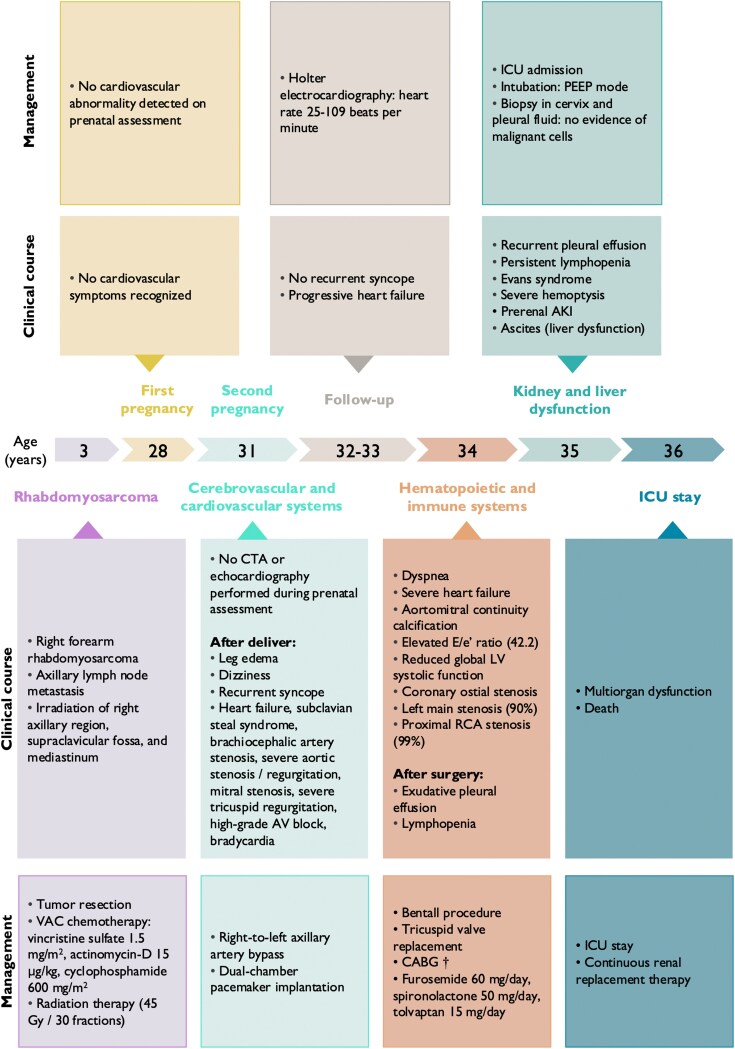


## Case presentation

A 31-year-old woman presented with recurrent syncopes. She had a history of childhood rhabdomyosarcoma of the right forearm, for which she underwent resection, chemotherapy, and radiation therapy at a total dose of 45 Gy in 30 fractions at 3 years of age (*Summary Figure*). The irradiated fields included the right axillary region, right supraclavicular fossa, and right mediastinum because of biopsy-proven axillary lymph node metastasis. After completing cancer therapy, she underwent annual follow-ups until she was 10 years old and remained asymptomatic for many years thereafter.

She became pregnant for the first time at 28 years of age and for the second time at 31 years. No cardiovascular abnormalities were detected during routine prenatal assessments, and neither computed tomography angiography nor echocardiography was performed during these pregnancies. After her second delivery, she developed leg oedema and complained of dizziness. After discharge, she experienced syncope while driving, followed by two additional syncopal episodes within a few months of discharge.

She was referred to our hospital for further evaluation, which revealed multiple concomitant cardiovascular abnormalities, including heart failure, subclavian steal syndrome (*Figure 1*), ascending aortic stenosis (*Figures 2* and *3*), severe aortic valve stenosis with moderate regurgitation, mitral valve stenosis, severe tricuspid regurgitation, high-grade atrioventricular block, and bradycardia due to autonomic dysfunction (*Figure 4*). Doppler carotid ultrasound showed calcification and atherosclerosis at the origin of the right common carotid artery, with circumferential thickening of the intima-media complex and 73.6% stenosis. Given the spectrum of cardiovascular abnormalities in areas suspected to have been irradiated, radiation-induced cardiovascular disease was diagnosed in this patient. The differential diagnosis included secondary malignancies, such as cardiac rhabdomyosarcoma.

**Figure 1:**
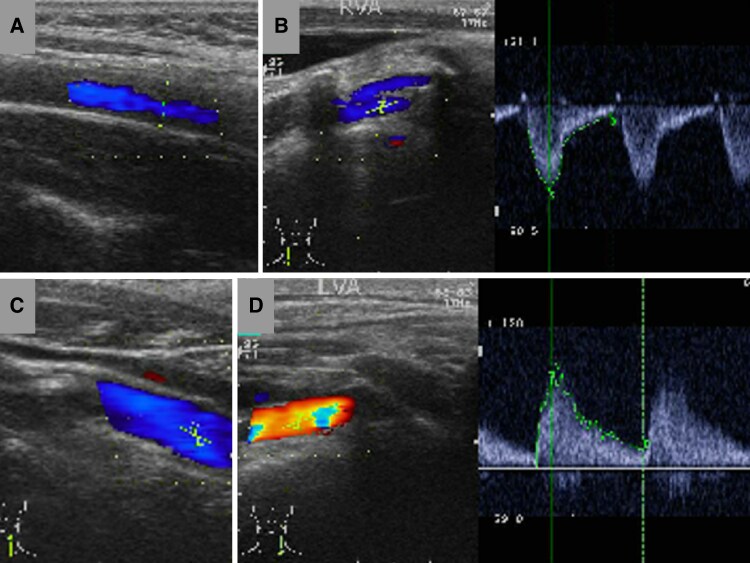
Vertebral artery Doppler ultrasonography. (*A*) and (*B*) Right vertebral artery in the longitudinal (*A*) and transverse (*B*) views. Panel *A* shows a narrowed right vertebral artery, and panel B demonstrates retrograde flow on spectral Doppler, consistent with the subclavian steal syndrome. (*C*) and (*D*) Left vertebral artery in the longitudinal (*C*) and transverse (*D*) views. Panel *D* shows the preserved antegrade flow on spectral Doppler.

**Figure 2:**
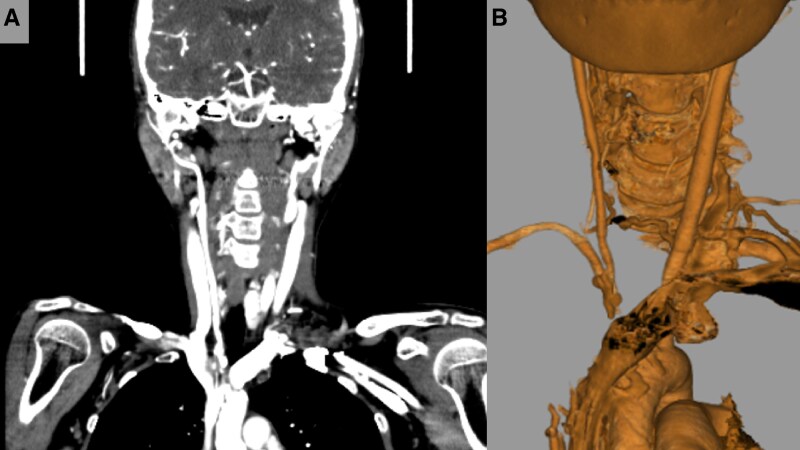
Computed tomography angiography suggestive of asymmetric vascular growth ipsilateral to the main irradiated field. (*A*) Two-dimensional computed tomography angiography showing smaller calibres of the right-sided arterial vasculature. (*B*) Three-dimensional computed tomography angiography reconstruction showing stenosis at the origin of the brachiocephalic artery, with smaller calibres of the brachiocephalic artery, right subclavian artery and right common carotid artery.

**Figure 3:**
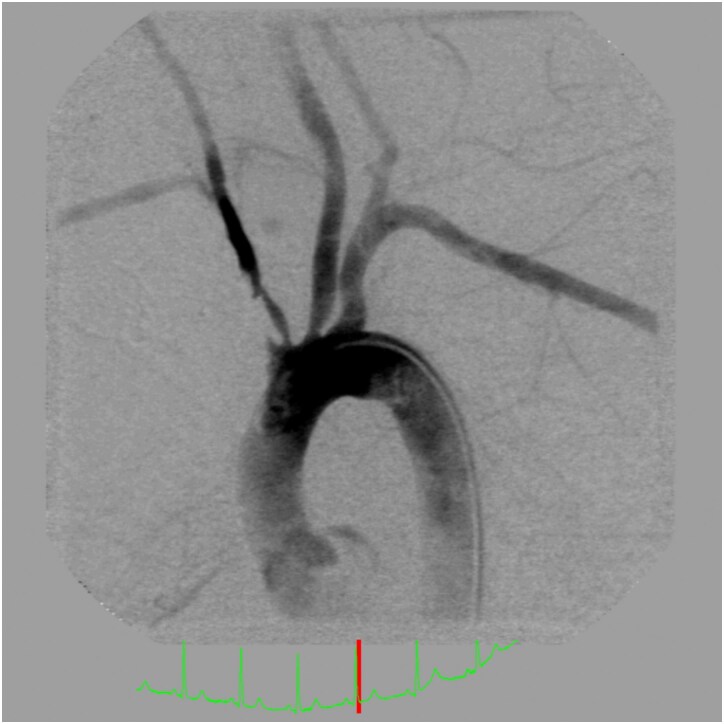
Aortic arch angiography showing stenosis of the brachiocephalic artery.

**Figure 4:**
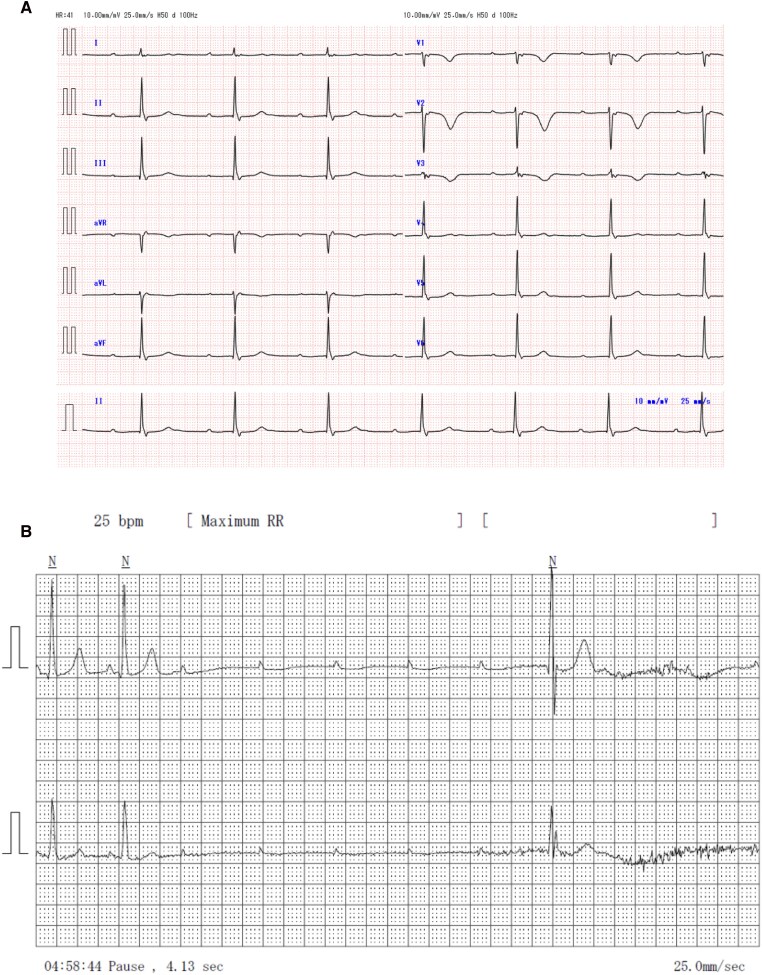
Electrocardiographic findings. (*A*) Twelve-lead electrocardiogram showing bradycardia. (*B*) Holter rhythm strip demonstrating bradycardia with a 4.3-s pause, suggestive of a high-grade atrioventricular block.

A right-to-left axillary artery bypass was performed to treat the focal cerebral hypoperfusion. A permanent dual-chamber pacemaker was implanted to treat the atrioventricular block and bradycardia. After these interventions, no further syncope was recorded during 2 years of follow-up. However, the patient’s heart failure continued to progress.

Two years after the first intervention, at 33 years of age, she was hospitalized for severe heart failure with dyspnoea. Further evaluation revealed worsening valvular heart disease, aortomitral continuity calcification (*Figure 5*), coronary ostial stenosis caused by calcification, and severe coronary artery stenosis, including 90% stenosis of the left main coronary artery and 99% stenosis of the proximal right coronary artery. Therefore, open surgery in a hostile chest was performed, including the Bentall procedure, tricuspid valve replacement, and coronary artery bypass grafting (CABG).

**Figure 5:**
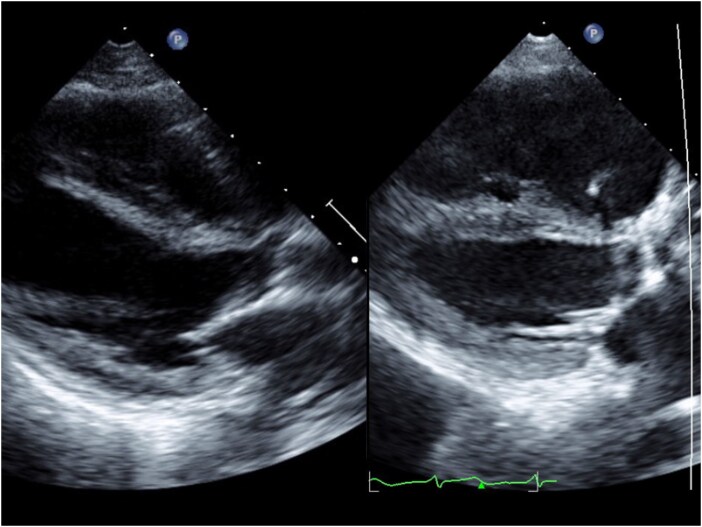
Echocardiographic findings. Left: Admission echocardiography. Right: Echocardiography performed 2 years later, showing more prominent aortomitral continuity calcification and pericardial thickening than in the left image. At this assessment, an elevated E/e’ ratio of 42.2 and reduced global left ventricular systolic function were also observed. E/e’, ratio of the early diastolic mitral inflow velocity (*E*) to early diastolic mitral annular tissue velocity (e’).

After surgery, her symptoms did not improve; instead, they progressed, mainly with postoperative pleural effusion and lymphopenia. The pleural effusion required combination diuretic therapy and was classified as exudative according to Light’s criteria.^2^ The patient was discharged 1 month after the second intervention. However, she required several readmissions due to recurrent pleural effusion. Diuretic therapy led to prerenal acute kidney injury.

During follow-up, lymphopenia persisted, and Evans syndrome was diagnosed 8 months after discharge. Over the following 10 months, her condition worsened, with shortness of breath, ascites due to liver dysfunction, severe haemoptysis, and pre-shock symptoms requiring intubation. However, the source of bleeding could not be identified as alveolar, tracheal, or gastrointestinal. During intensive care, organ dysfunction progressed, particularly in the kidneys and liver, and the patient died of multiorgan dysfunction.

## Discussion

The patient experienced radiation-induced late effects, particularly involving the haematopoietic and immune systems, which have a rapid cell turnover, and the cerebrovascular and cardiovascular systems, which are slow-turnover tissues. These effects are not limited to cell killing but also include tissue reactions that may become apparent decades after exposure.^1^

Asymmetric vascular growth is an example of this process. In the acute phase, radiation-induced cell injury may have reduced tissue regeneration in the right-sided arterial vasculature ipsilateral to the main irradiated field, whereas chronic vascular stenosis and intimal fibrosis developed later on. After radiation exposure, endothelial injury induces the release of inflammatory cytokines, allowing leukocyte infiltration into the vessel wall. Oxidative stress and deoxyribonucleic acid damage promote cellular senescence and impair the regenerative capacity of healthy stem cells.^1,3^ Together with tumour resection involving surrounding parenchymal tissue, these processes may have partly contributed to the atrophy of the ipsilateral vasculature.^4^ In parallel, reactive oxygen and nitrogen species generated within irradiated tissues alter proteins, lipids, carbohydrates, and other molecules, thereby activating secondary signalling pathways. Fibrosis is not caused by cell death but by premature senescence and accelerated postmitotic differentiation, which lead to excessive collagen production by irradiated mesenchymal cells, including fibroblasts, myofibroblasts, and smooth muscle cells.^1^ This delayed fibrotic process may explain why her first episode of syncope was recognized in her 30 s rather than earlier. In this case, cumulative collagen deposition and fibrosis were also suggested by typical findings, such as aortomitral continuity calcification,^3^ and by less commonly reported manifestations, including atrioventricular block and autonomic dysfunction.^1^

The clinical course may not be explained solely by radiation-induced effects but also by complications arising from the challenging treatment of this complex case. Two events appeared to markedly accelerate the clinical deterioration: her second delivery and open-chest surgery. A meta-analysis reported that patients with a history of cancer therapeutics-related cardiac dysfunction had an incidence of heart failure or left ventricular dysfunction of 28.4%, with an odds ratio of 47.4.^5^ This finding suggests that pregnancy-related haemodynamic stress may have unmasked underlying cardiovascular vulnerability.^6^ In addition, despite low perioperative mortality, mid- and long-term morbidity and mortality remain high in patients with a history of radiation therapy, even when conventional cardiac surgery or CABG is technically successful.^7,8^ The lymphopenia observed after open-chest surgery was reminiscent of the severe lymphopenia that occurs after lethal irradiation, a characteristic feature of haematopoietic syndrome. Subsequent lymphopenia, neutropenia, and thrombocytopenia may reflect injury to haematopoietic stem cells and progenitor cells, together with apoptosis-related acute cell death.^1^ Furthermore, radiation-induced injury and abnormal wound healing share common mechanisms, including cytokine cascades and chemokine-mediated recruitment of inflammatory cells, which further promote collagen deposition.^4,9,10^ Persistent inflammation may impair immune regulation and could have contributed to the development of Evans syndrome in this case. In addition, refractory postoperative pleural effusion has been reported in patients with cancer,^11,12^ warranting further investigation. Antibiotics have also been reported to enhance radiation responses.^1^

Given the difficulty in managing these patients, structured long-term surveillance and careful pre-intervention assessments should be emphasized. With advances in radiation therapy techniques aimed at reducing exposure to organ at risk, together with prone positioning and breathing techniques to reduce mean organ radiation dose, the risk of radiation-induced disease may be mitigated.^10,13^ Screening frequency should be determined according to the total radiation dose or mean organ dose, as well as the age at initiation of radiation therapy.^14^

## Conclusions

Late effects may become apparent long after the completion of radiation therapy and may involve multiple cardiac structural and functional abnormalities. Screening should follow guideline recommendations based on patient risk stratification to allow timely treatment and interventions.

## Data Availability

Data for this article are available from the corresponding author upon request. No new data were generated or analysed in support of this study.

## Lead author biography

Tuan Hoang Nguyen is a PhD candidate in Cardiovascular Medicine at the University of Yamanashi. He holds a master’s degree in Internal Medicine from the University of Medicine and Pharmacy at Ho Chi Minh City, Vietnam. His research interests include lipid metabolism, inflammation, heart failure, and cardiovascular prevention.

## Notes

**Consent:** The authors confirm that written informed consent for the submission and publication of this case report, including the images and associated text, was obtained from the patient in accordance with the Committee on Publication Ethics (COPE) guidance.
